# Supplementary material for: Solid Phase Synthesis and TAR RNA-Binding Activity of Nucleopeptides Containing Nucleobases Linked to the Side Chains via 1,4-Linked-1,2,3-triazole
Source: Biomedicines. 2024 Mar 3;12(3):570. doi: 10.3390/biomedicines12030570 (PMC10968536; doi:10.3390/biomedicines12030570)
Supplement: Supplementary file 1 [file biomedicines-12-00570-s001.zip › biomedicines-2809758-supplementary.pdf]

biomedicines

MDPI

Supplement data

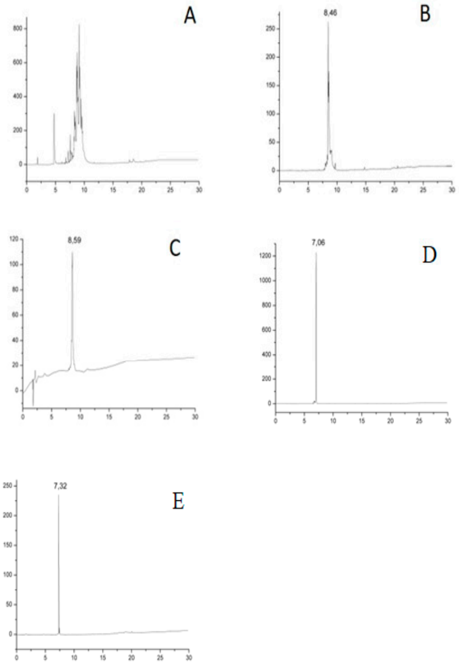

Figure S1. Analytical RP-HPLC analysis of HalTzl$_{TCCCAG}$ synthesis and purification. (A) Crude HalTzl$_{TCCCAG}$ after resin cleavage procedure, (B) crude HalTzl$_{TCCCAG}$ after dissolving in 1M K_2CO_3/MeOH solution for 1h, (C) HalTzl$_{TCCCAG}$ after semi-preparative RP-HPLC purification, (D) HalTzl$_{AAA}$ after semi-preparative RP-HPLC purification, (E) . HalTzl$_{AGA}$ after semi-preparative RP-HPLC purification. Detection was carried out at 254 nm.
